# Editorial: Diabetes and aging

**DOI:** 10.3389/fendo.2022.1091358

**Published:** 2022-11-18

**Authors:** Helena Cristina de Lima Barbosa

**Affiliations:** Obesity and Comorbidities Research Center, University of Campinas, UNICAMP, Campinas, Sao Paulo, Brazil

**Keywords:** diabetes, aging, elderly, T2DM, insulin

Diabetes mellitus is one of the major cause of morbidity and mortality, and it is a major risk factor for early onset of several disfunction. The present Research Topic has been designed to publish original articles and reviews highlighting recent advances in our understanding of diabetes and the importance of glycemic control in elderly. Emphasis has been given on the underlying molecular mechanisms, the new technologies that have been introduced to facilitate early diagnosis or prevention, and the new potential therapies for the associated complications.

In a paper of this Research Topic entitled “*LC-MS-Based Untargeted Metabolomics Reveals Early Biomarkers in STZ-Induced Diabetic Rats With Cognitive Impairment*”, Chen et al. performed a non-targeted metabolomics approach based on liquid chromatography-mass spectrometry (LC-MS), to screen out the serum biomarkers of diabetic mild cognitive impairment (DMMCI) in rats. Differentially expressed metabolites could provide a novel strategy for the early diagnosis of DMMCI and give new insights into the pathophysiological changes and molecular mechanisms of disease in the future. In this study, the authors used a combination of low-dose streptozotocin and a high-fat diet to establish a rat model mimicking human the T2DM model, and observed its cognitive deficit. They showed that an LC-MS-based metabolomics technology has potential value in identifying DMMCI biomarkers for the early detection and provides a novel avenue for effective therapeutic intervention in DCD, as detected in serum, sphingolipid (SP) metabolism, tryptophan (Trp) metabolism, glycerophospholipid (GP) metabolism, these metabolites may be used as the most critical biomarkers for early diagnosis of DMMCI.

In the article “*Aging Reduces Insulin Clearance in Mice*” Marmentini et al. investigated whether the effects of aging upon hepatic insulin clearance were related to changes in the carcinoembryonic antigen-related cell adhesion molecule 1 (CEACAM1) and insulin-degrading enzyme (IDE) expression, as well as IDE activity, in the liver of old mice. The authors pointed that although several studies have considered IDE as the major enzyme involved with hepatic insulin clearance, recent studies suggest that other molecular mechanisms must be more important to modulate hepatic insulin clearance, such as CEACAM1 expression. To achieve that, the authors evaluated the glucose homeostasis, insulin secretion and hepatic insulin clearance in 3- and 18-month-old mice. Insulin clearance reduces with age and this may contribute to age-related hyperinsulinemia. Although previous studies suggest that IDE is not involved in the modulation of hepatic insulin clearance, in control and obese mice, Marmentini et al. suggest that during aging this enzyme might have a role in this modulation, as well as, the CEACAM1. Thus, the authors suggested that to investigate the molecular mechanisms whereby aging reduces IDE and CEACAM1 function in the liver might be helpful to understand how insulin clearance is affected by age.

In another article, entitled “*Glycemic Dysregulations Are Associated With Worsening Cognitive Function in Older Participants at High Risk of Cardiovascular Disease: Two-Year Follow-up in the PREDIMED-Plus Study*” the authors aimed to examine 2-year associations between baseline diabetes/glycemic status and changes in cognitive function in older participants at high risk of cardiovascular disease. As pointed, meta-analyses and longitudinal studies of population-based cohorts have shown an increased risk of cognitive dysfunction in people with metabolic syndrome, prediabetes and diabetes. Thus, Gómez-Martínez et al. evaluated longitudinal associations between glycemic status (diabetes status, control/treatment, and related biomarkers) and cognitive decline and impairment using 2 years of follow-up data, within the framework of the PREDIMED-Plus, which is a multicenter, randomized, parallel-group clinical trial conducted in Spain for primary cardiovascular disease prevention. The author pointed that the work is the first prospective study investigating associations between glycemic status (diabetes status/control/treatment, and HOMA-IR and HbA1c biomarkers) and cognitive function in a large cohort of older adults at risk high cardiovascular disease in a short period. Eligible participants were community-dwelling adults with overweight/obesity (27≤ BMI <40 kg/m2) who met at least three criteria of metabolic syndrome. The study suggested larger risk of cognitive decline in participants with type 2 diabetes. Also, they showed that, compared to participants without diabetes, those with diabetes had a borderline increased risk of developing cognitive impairment, even when the period of follow-up was only 2 years. Besides, no associations between metformin treatment and cognition were observed by the authors, as well this was not observed for IDDP-4 or sulfonylureas use. However, insulin-treated participants showed larger cognitive decline than those not treated with insulin. Thus, Gómez-Martínez et al. concluded that several glycemic dysregulations and insulin treatment were associated with greater cognitive decline in older individuals with overweight/obesity at high cardiovascular disease risk in a short time period, pointing the clinical relevance to assess novel effective strategies at the initial stages of diabetes-related alterations.

In their study titled “*Comparative Cardiovascular Outcomes of SGLT2 Inhibitors in Type 2 Diabetes Mellitus: A Network Meta-Analysis of Randomized Controlled Trials*”, Jiang et al. performed an network meta-analysis (NMA) of randomized controlled trials (RCTs) for the first time to explore cardiovascular outcomes of different kind and dosages of sodium-glucose cotransport-2 (SGLT2) inhibitors in T2DM patients, including dapagliflozin 2.5mg/5mg/10mg, empagliflozin 10mg/25mg, and canagliflozin 100mg/300mg. The authors searched for studies to compare the therapeutic effects of different SGLT2 inhibitors in T2DM patients, using Cochrane Library, PubMed, and Embase databases. As suggested by the authors, empagliflozin 10mg/25mg, and canagliflozin 100mg was associated with significantly lower risks of all-cause mortality compared with placebo, according to NMA. Their study also suggested that empagliflozin 10mg/25mg was leaded to significantly lower risks of all-cause mortality compared with dapagliflozin 10mg. Dapagliflozin 10mg, empagliflozin 10mg and 25mg displayed the lower risks for cardiovascular events compared with placebo. In addition, they pointed that canagliflozin 100/300mg showed significantly higher risks of cardiovascular events compared with empagliflozin 10mg/25mg according to NMA. Moreover, their analysis suggested that treatment with canagliflozin 100/300mg were associated with significantly increased risks of volume depletion compared with placebo by NMA. The authors concluded that empagliflozin 10mg/25mg once daily might be better than other SGLT2 inhibitors with low risks of all-cause mortality and cardiovascular events in patients with T2DM suggesting the need for *ad hoc* RCTs.

As part of this Research Topic, also figured the article “*Hyperglycemia and Physical Impairment in Frail Hypertensive Older Adults*”, where Pansini et al. aimed at investigating the impact of hyperglycemia (HG) on physical impairment in frailty, as HG is frequently observed in frail older adults, and represents an independent predictor of worst outcomes, with or without diabetes mellitus. The authors mentioned that the results refer to a frail hypertensive population of older adults, in which physical performance affects functional decline, loss of independence, and cognitive impairment. Their interesting study suggested that HG drives physical impairment independently of DM and the authors speculated that glycemic control appears to be the best way to attempt to reverse physical impairment, with or without DM. Pansini et al. pointed that the study population was relatively small, therefore, further studies are necessary to confirm their results, ideally in large randomized trials.

In the article “*Association Between Long-Term HbA1c Variability and Functional Limitation in Individuals Aged Over 50 Years: A Retrospective Cohort Study*” Shao et al. explored the longitudinal association between long-term glycemic variability, represented by visit-to-visit HbA1c variability and functional limitations. They analyzed adults aged over 50 years who participated in the 2006 to 2016 waves of the Health and Retirement Study. The authors pointed that limitation of physical functioning threatens independence and is an independent risk factor for impaired quality of life, institutionalization, further functional decline, and premature mortality in older adults. They indicated that the association between diabetes and functional limitation and disability is well documented, but mean HbA1c provides incomplete information regarding glycemic variability. Thus, the authors explored whether glycemic variability in individuals without diabetes is an independent risk factor for functional limitation, which is currently unknown, using data from the 2006 to 2016 waves of the Health and Retirement Study. Shao et al. found that HbA1c variability was associated with more difficulties in functional activities over time, indicating that HbA1c variability was a superior predictor of functional decline over mean HbA1c. Their results showed the association between glycemic variability, as measured by variability score in visit-to-visit HbA1c over time, and the number of physical functioning difficulties independent of mean HbA1c in individuals aged over 50 years. The authors also pointed that more trials are needed to establish glycemic variability as an independent risk factor for functional decline and diabetes complications, and regarding the importance to confirm whether strategies to reduce glycemic variability in HbA1c can effectively reduce the incidence or progression of physical functioning impairment.

Turning their attention to the complex link between type 2 diabetes, cognition, and neurovascular coupling, Barloese et al. worked in the review “*Neurovascular Coupling in Type 2 Diabetes With Cognitive Decline. A Narrative Review of Neuroimaging Findings and Their Pathophysiological Implications*”. In the article they discuss how the disease-related pathology changes neurovascular coupling (NVC) in the brain from the organ to the cellular level. The authors pointed that a clinical manifestation cognitive impairment or so-called diabetic “cogno-pathy” is receiving increasing attention. As mentioned by the authors, the identification of neurovascular abnormalities that are attributable to diabetes and precede structural and clinical changes, holds the potential to guide personalized preventive interventions. In this line, Barloese et al. focused on how NVC is impaired by T2DM and how it is possible to measure T2DM-related neurovascular dysfunction in humans. The authors pointed modalities that are used to measure NVC in humans, and that each of them has its limitations, however, there is converging evidence for an independent effect of the T2DM-state on NVC with cognitive decline as a possible progressing clinical correlate. Thus, the authors suggest the importance of early detection of impaired NVC in T2DM patients and preventive treatment before irreversible damage occurs.

Kim et al. in their paper titled “*Tolerability and Effectiveness of Switching to Dulaglutide in Patients With Type 2 Diabetes Inadequately Controlled With Insulin Therapy*” conducted a retrospective, observational study, to investigate whether switching to dulaglutide, a GLP-1 receptor agonist, would improve glycemic control of patients with T2DM inadequately controlled with conventional insulin treatment. As the authors pointed, it is common the use of insulin as an adjunct to oral hypoglycemic agents (OHAs). Thus, they analyzed the human subjects’ medical record and laboratory data of patients with T2DM whose HbA1c levels were 7.6% or higher when treatment was switched from insulin to dulaglutide. Replacing insulin therapy with a combination of a GLP-1 receptor agonist and OHAs could be effective in patients with uncontrolled T2DM receiving insulin therapy. They showed that 20 patients with T2DM (approximately 14.5%) could not tolerate or did not prefer weekly dulaglutide administrations (reasons included cost, gastrointestinal side effects, dissatisfaction with the drug), and 56 (approximately 40.6%) could successfully discontinue insulin and use either weekly dulaglutide or OHAs and presented glycemic effectiveness after the switch. The mean HbA1c value in that group significantly reduced from 8.7% to 7.8%, and of the 56 patients, 23 (16.7%) patients could completely cease all injection therapies including dulaglutide and maintained stable glycemia over the 6-month period. They also found that older age, a higher dose of insulin at the time of switching to dulaglutide, and a low level of postprandial glucose were significant predictive factors for insulin resumption after switching from insulin to weekly dulaglutide. Kim et al. concluded that dulaglutide can be used for glycemic control in patients with T2DM with glucose levels inadequately controlled by insulin regimens. The authors pointed several limitation of the work, as was an uncontrolled, open-label, longitudinal, retrospective study, which is limited in its applicability and clinical relevance to generalization and broader clinical practice. Besides, the short follow-up period is an other limitation of their study. However, they highlight that the findings show the natural results of real-world practice that did not involve any interventions.

In a cross-sectional study, tilted “*Influence of circulating nesfatin-1, GSH and SOD on insulin secretion in the development of T2DM*”, Huang et al. aimed to evaluate the correlation of nesfatin-1, GSH and SOD levels with beta cell insulin secretion and their influence on insulin secretion in the development of T2DM. They analyzed serum levels of nesfatin-1, GSH and SOD from 75 patients with T2DM, 67 with prediabetes and 37 healthy participants, that were recruited in this study. The author pointed that in face of multiple explanations proposed in the development of T2DM, oxidative stress is considered to be pivotal in this process, and evaluation of glutathione (GSH) or superoxide dismutase (SOD) are important indicative of oxidative stress. Also, they highlight nesfatin-1, a newly identified peptide with 82 amino acids, that has been found to be functional in anti-inflammation and antioxidation. Thus, evaluating important factors involving insulin secretion in the development of T2DM, the authors aimed to provide new ideas for forthcoming investigations on the roles of these factors in pathogenesis of T2DM. They divided T2DM and prediabetes patients into subgroups by HOMA-β with the cut-off value of 62.9 for male and 60.6 for female. Also, to assess whether beta cell insulin secretion varies in prediabetes, three subgroups of impaired fasting glucose (IFG), impaired glucose tolerance (IGT) and IFG combined IGT were divided, according to the American Diabetes Association classification, and their HOMA-β values were compared. In a scenario, where there were no significant differences in gender, age and BMI among the three studied groups, the authors showed that serum GSH levels in T2DM were significantly reduced than that in prediabetes or the control, and this significant reduction was also confirmed in prediabetes vs. the control. Besides, further comparisons revealed that the difference of GSH levels among prediabetes subgroups of IGT, IFG and IFG+IGT was insignificant. Also, they showed that GSH levels in either subgroup of T2DM or prediabetes with impaired HOMA-β values were overwhelmingly dropped, in contrast to the counterparts with normal HOMA-β. Besides, they found that SOD levels in T2DM and prediabetes were remarkably decreased compared with the healthy control, and also a significant reduction of the SOD level in T2DM vs. prediabetes. Moreover, they observed that serum SOD levels in subgroup of IFG or IFG combined IGT displayed a marked reduction compared to the IGT subgroup. Their results of GSH and SOD reduction in T2DM and prediabetes suggest that in the condition of T2DM or prediabetes, the anti-oxidation capacity in the body may be partly damaged. In that way, they suggest that fortifying the antioxidative defense system of the patients with prediabetes may help regress or alleviate the progression of the disease toward T2DM. In addition, they found that serum nesfatin-1 levels in T2DM were obviously reduced compared to that in prediabetes or healthy subjects, and his reduction still presented when they compared prediabetes to the control. Besides, they found that difference of serum nesftain-1 levels in IGT were insignificant compared to either in IFG or in IFG+IGT, and that nesfatin-1 levels were significantly correlated with GSH and SOD, indicating a high probability of nesfatin-1 exerting antioxidative effects in the development of T2DM. In conclusion, despite the limitations of the study, as pointed by the authors, Huang et al. study managed to identify the correlation of nesfatin-1, GSH and SOD levels with beta cell dysfunction in T2DM, implicating their roles in beta cell toxicity as a result of oxidative stress.

In the study “*Relationship between physical performance and mild cognitive impairment in elderly hemodialysis patients is modified by the presence of diabetes: A multicenter cross-sectional study*”, Zhao et al. aimed to explore the relationship between physical performance and mild cognitive impairment (MCI) in elderly hemodialysis patients with and without diabetes. They hypothesized that the presence of diabetes would lead to poorer physical performance and high prevalence of MCI, and different conditions may influence the association between physical performance and MCI, and also investigated the association between physical performance and specific cognitive functions in the presence or absence of T2DM in hemodialysis patients. To achieve that, Zhao et al. performed a multicenter cross-sectional study recruiting patients, aged 60 years or older, who underwent hemodialysis in dialysis units. They formed four groups: non-diabetes non-MCI, non-diabetes MCI, Diabetes non-MCI, and Diabetes MCI, a total of 396 patients. The authors found that diabetic hemodialysis patients with MCI performed worse mobility than the non-diabetes group, and that, whether compared with MCI in the non-diabetes group or non-MCI in the diabetes group, diabetic patients with MCI have poor mobility. Also, they found that the prevalence of MCI in diabetic hemodialysis patients was high (20.6%), thus they pointed that diabetes in end-stage renal disease patients receiving hemodialysis may be an important risk factor for the development of MCI. Besides, in face of a significant interaction found between mobility and diabetes in hemodialysis patients in the study, they suggested that poor physical performance due to diabetes may be an important risk factor for the development of MCI. However, they also pointed the need of future studies focused on the different cognition changes in the weak physical population, in more well-designed cohort studies to verify the relationship between physical performance and different cognitive functions. In conclusion, their study study provides considerations for physicians that poor mobility in diabetic hemodialysis patients are more associated with MCI.

In the last study, titled “*The clinical characteristics of Chinese elderly patients with different durations of type 2 diabetes mellitus*” Yu et al. explored the clinical characteristics among 3840 elderly (aged ≥60 years) patients, diabetes duration and the comprehensive management of T2DM as well as diabetic vascular complications in Chinese elderly patients with T2DM. The authors studied 972, 896, 875 and 1097 patients, that were respectively divided into four groups, according to diabetic duration: < 1 year (Group 1), 1~5 years (Group 2), 5~10 years (Group 3), and ≥ 10 years (Group 4). They found that compared to group 1, the level of HbA1c was significantly higher in group 4, but was significantly lower in group 2 and group 3. Also, they observed that group 4 had a significantly higher control rate of total cholesterol (TC) when compared with groups 1, similarly for the control rates of low density lipoprotein cholesterol (LDL-C). Besides, patients of group 4 were more likely to be higher control rate of triglyceride (TG) and body mass index (BMI) when compared with other groups. They also found that elderly T2DM patients with a duration of diabetes of ≥ 10 years were more likely to achieve the comprehensive control targets for TC, LDL-C and TG, while elderly T2DM patients with a duration of diabetes of 1~5 years were more likely to achieve the HbA1c control target than elderly T2DM patients with a duration of diabetes of < 1 year. The authors pointed that the higher control rates for TC, LDL-C, TG and BMI were observed in elderly T2DM patients with a duration of diabetes of ≥ 10 years than that in patients who had a duration of diabetes less than 1 year. Their study also suggest that elderly T2DM patients with a duration of diabetes of 5~10 years or ≥ 10 years were more likely to develop diabetic macrovascular complications than those with a duration of diabetes of <1 year. In addition, they indicated that the duration of diabetes was significantly associated with microvascular complications. Yu et al. concluded that the clinical characteristics of elderly patients with T2DM in different durations of diabetes are different.

## Author contributions

The author confirms being the sole contributor of this work and has approved it for publication.

## Funding

This study was supported by the Fundação de Amparo à Pesquisa do Estado de São Paulo (FAPESP).

## Acknowledgments

I wish to thank all the authors who submitted their study for consideration for the present Research Topic and the reviewers who evaluated the papers. Their efforts and valuable contribution were very special, and essentially turned this Research Topic possible.

## Conflict of interest

The author declares that the research was conducted in the absence of any commercial or financial relationships that could be construed as a potential conflict of interest.

## Publisher’s note

All claims expressed in this article are solely those of the authors and do not necessarily represent those of their affiliated organizations, or those of the publisher, the editors and the reviewers. Any product that may be evaluated in this article, or claim that may be made by its manufacturer, is not guaranteed or endorsed by the publisher.

